# Towards Accurate Microwave Characterization of Tissues: Sensing Depth Analysis of Open-Ended Coaxial Probes with Ex Vivo Rat Breast and Skin Tissues

**DOI:** 10.3390/diagnostics11020338

**Published:** 2021-02-18

**Authors:** Cemanur Aydinalp, Sulayman Joof, Tuba Yilmaz

**Affiliations:** Department of Electronics and Communication Engineering, Istanbul Technical University, 34469 Istanbul, Turkey; aydinalp16@itu.edu.tr (C.A.); joof@itu.edu.tr (S.J.)

**Keywords:** open-ended coaxial probes, sensing depth analysis, heterogeneity of biological tissues, rat’s tissue, animal experiment

## Abstract

Dielectric properties of biological materials are commonly characterized with open-ended coaxial probes due to the broadband and non-destructive measurement capabilities. Recently, potential diagnostics applications of the technique have been investigated. Although the technique can successfully classify the tissues with different dielectric properties, the classification accuracy can be improved for tissues with similar dielectric properties. Increase in classification accuracy can be achieved by addressing the error sources. One well-known error source contributing to low measurement accuracy is tissue heterogeneity. To mitigate this error source, there is a need define the probe sensing depth. Such knowledge can enable application-specific probe selection or design. The sensing depth can also be used as an input to the classification algorithms which can potentially improve the tissue classification accuracy. Towards this goal, this work investigates the sensing depth of a commercially available 2.2 mm aperture diameter probe with double-layered configurations using ex vivo rat breast and skin tissues. It was concluded that the dielectric property contrast between the heterogeneous tissue components has an effect on the sensing depth. Also, a membrane layer (between 0.4–0.8 mm thickness) on the rat wet skin tissue and breast tissue will potentially affect the dielectric property measurement results by 52% to 84%.

## 1. Introduction

Interaction of electromagnetic fields with biological tissues has been a research topic for almost a century partly due to ever increasing pervasiveness of such signals in the environment [1,2,3]. Dielectric properties govern the interaction between the biological tissue and the electromagnetic waves; therefore, the accurate knowledge of dielectric properties is crucial for modeling of electromagnetic wave and biological tissue interaction. Open-ended coaxial probes have been widely used for dielectric property characterization of biological tissues [4,5,6]. Due to the potential applications of electromagnetics in breast cancer research such as microwave breast imaging and breast cancer hyperthermia, the dielectric properties of breast tissue have been a research interest. In [7], the dielectric properties of normal, malignant and benign breast tissues were reported from 0.5 to 20 GHz using the open-ended coaxial probe technique. Measurements carried out with ex vivo human breast tissues from 0.5 to 50 GHz were reported in [8]. These studies formed the dielectric property database for the microwave breast cancer research on imaging and hyperthermia applications. Recently, in vivo rat breast tissue dielectric properties have been explored to unveil other diagnostic applications such as potential use of open-ended coaxial probes for biopsy purposes [9]. It should be noted that the emerging diagnostic applications of the technique have also revealed the need to measure the experiment animal breast tissue dielectric properties. Although promising results for categorizing the high dielectric property difference tissues were obtained [7,8,10,11,12], there is still a need to improve the classification accuracy for tissues with low dielectric property difference. The technique is known to suffer from high measurement error and low measurement repeatability. The error can stem from the tissue heterogeneity or equipment degradation [13]. Equipment related errors are mitigated by improving the calibration methods. However, it was shown that the large error margins tend to mostly emanate from tissue heterogeneity [14]. To realize practical applications of the probe, there is a need to investigate the probe sensing depth where the sensing depth can either be used to choose the probe type or it can be used as an input for machine or deep learning algorithms for accurate characterization of the tissue under investigation.

Several studies have been reported on the open-ended coaxial probe sensing depth definition to overcome the tissue heterogeneity related errors. These studies used different terminologies such as effective penetration depth, sensing volume, histology region and corresponding definitions for the used terminologies [15,16,17,18]. For example, in [16], sensing volume of open-ended coaxial probe with 2.2 mm aperture diameter was investigated using pure alcohols and de-ionized water. Required sample thickness for 2.2 mm probe was determined as 1.5 mm for the samples. Similarly, in [17], sensing volume of 2.2 mm aperture diameter probe was determined as 200 to 400 microns where the tissue heterogeneity was modeled using double-layered Teflon and de-ionized water configuration. In [15], the effective penetration depth characterized using previously introduced Teflon and de-ionized water configuration was used to measure the penetration depth with 2.2 mm aperture diameter open-ended coaxial probe. The definition used for the reported experiment in [15] was 20% change in measured permittivity values and the penetration depth was given as 0.28 mm. The percent change was determined through a linearly extrapolated line. In another study [18], the histology region was defined as the depth at which the measured results can reflect the changes in the tissue sample. When determining the changes, the measurement uncertainty was considered. The analysis in the reported work was carried out with six different sample configurations, two of which included ex vivo porcine tissue. Determined histology region for the 2.2 mm aperture probe was varied from 1.20 to 3.65 mm based on the frequency and the material under test.

Although the reported studies give valuable insights, the sensing depth of the probe with low dielectric property difference samples have not been explored. Mostly high dielectric property difference scenarios, such as Teflon and de-ionized water or muscle and fat were used for heterogeneity representation. However, the heterogeneity can have varying dielectric property discrepancies in a realistic setting. One example is the difference between breast benign and malignant tumor tissues reported as 10% [7]. Therefore, this study explores open-ended coaxial probe sensing depth with samples with varying heterogeneity in an attempt to understand the effect of dielectric property difference on sensing depth. The heterogeneity was represented by using double-layered configurations composed with ex vivo rat breast tissue and oil also ex vivo rat skin tissue and triton X-100. Rat tissues were chosen due to common use of rat models for as experiment animals [19]. The experiments were conducted using commercially available and widely used 2.2 mm slim form open-ended coaxial probe. Unlike previously introduced specialized measurement tanks, a simple measurement setting was used. Sensing depth was determined by tracking five different measured dielectric property percent change values.

Remainder of this paper is arranged as follows: Section 2, background is given. In Section 3, materials and methods including experiment setup, sample configuration and measurement protocol are explained in detail. Section 4 presents results obtained from the experiments. In Section 5, findings are discussed in detail.

## 2. Background: Rat Tissue Characteristics

The skin is the most sizable organ, since it covers the entire surface area of the body. It mainly functions as a barrier against dehydration and microbial infection from the outside environment. The skin is comprised of various tissue types: (1) epidermis (epithelial cells), (2) dermis (collagen, fibroblasts, mast cells, nerves, blood vessels), (3) hypodermis (white adipose tissue), (4) panniculus carnosus muscle, and (5) adventitia (subcutaneous tissue) [19].

1. The epidermis contains one or two cell layers of cornified stratified squamous epithelium [20].
2. The dermis is a connective tissue that takes place beneath the epidermis and serves as a support with many blood vessels, lymphatic vessels and nerve fibers. Moreover, the hair follicles with their attendant sebaceous glands and the sweat glands are in the dermis [20].
3. The hypodermis is a layer of white adipose tissue (white fat) under the dermis. Adipose tissue is formed of adipocytes which are circular cells containing fat vacuoles. The hypodermal thickness is an important factor that shows the nutritional condition of the animal.
4. The panniculus carnosus is a skeletal muscle and can be described as the boundary between the dermal layers and the underlying adventia.
5. Subcutaneous tissue is a non-uniform collagenous tissue beneath the panniculus carnosus and contains fibroblasts, nerves, and blood vessels. It connects the skin to the skeletal muscle and provides the necessary elasticity and movements for the skin.

This intricate organ constitutes various adnexal structures such as modified sebaceous glands, eccrine and apocrine sweat glands. For instance, the mammary glands are a modified form of apocrine sweat gland. Since the mammary glands are formed from the epidermal precursors that develop downward during prenatal development, they are considered to be cutaneous adnexa.

**Figure 1:**
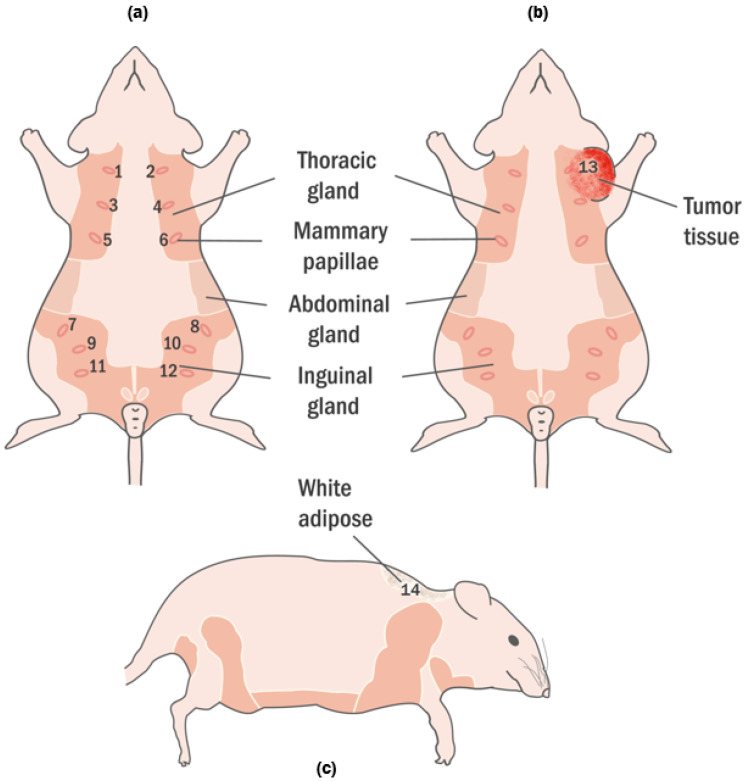
Healthy and tumor-bearing rats have been demonstrated. (**a**) Healthy rats are shown in the frontal plane with marked 12 nipples. The locations of the nipples are in the region of thoracic and inguinal. (**b**) The rat with tumor is shown in the frontal plane and tumor was marked with 13. The tumor according to the mammary tissues was formed around the 2nd and 4th tissue. (**c**) The healthy rat was shown in the sagittal plane. The white adipose, located on the nape, is marked 14.

Nipples are in the abdominal region as 6 pairs following lines that curve towards the midline. Generally, they are 2 mm in length; however, they grow to around 4 mm during lactation period [20]. Beneath the rat’s pelt, the mammary glands are placed within subcutaneous fat pads that can be divided into six mammary complexes: three pairs in the thoracic region and three pairs in the inguinal region as shown in Figure 1 [20]. They are branched tubular organs formed of one or two epithelial cell layers and are responsive to the hormonal and local epigenetic changes (adolescence, pregnancy, lactation, and senescence) [19]. It is essential to emphasize when the extensive rat mammary fat pads are completely developed, they extend from the inguinal area and base of the tail to the salivary gland region of the neck and around to the dorsum [19].

Dielectric property measurements of breast tissue, white adipose tissue, wet skin tissue and tumor tissue were performed to analyze the interior part of the pelt which has an intricate structure. The experiment was carried out with two female Sprague-Dawley rats obtained from Bogazici University, Center for Life Sciences and Technologies. To facilitate the forming of breast tumors, one animal (47 days old) was subjected to a single dose of carcinogenic solution that was prepared by dissolving 20 mg/kg 7,12-Dimethylbenz(a)anthracene (DMBA) in 1 mL olive oil in an ultrasound bath. Twenty-four hours prior to the application, the animal was prevented from accessing the food dispenser. The carcinogenic solution was applied via oral gavage and the animal was given a two-day rest period. The peritoneal area was checked for tumor formation by hand weekly. The resting conditions for the animals throughout the experiment were set as a 12 h light/dark cycle and free access to standard pellet food as well as tap water. The other animal was selected to be the control and was given a dummy solution of 1 mL olive oil via oral gavage, under the same conditions. Weight of the control and experiment animal during the experiments were 285 g and 230 g, respectively. Also, during the experiments, the ages of the control and experiment animal were 359 and 261 days old, respectively.

**Figure 2:**
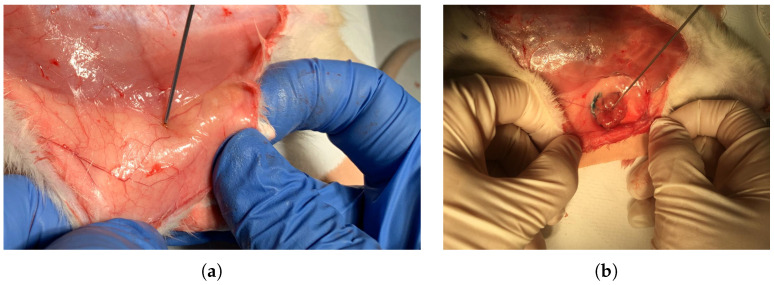
In vivo dielectric property measurements: (**a**) Measurement collected from the healthy breast tissue and (**b**) measurement of the malignant breast tumor tissue.

**Figure 3:**
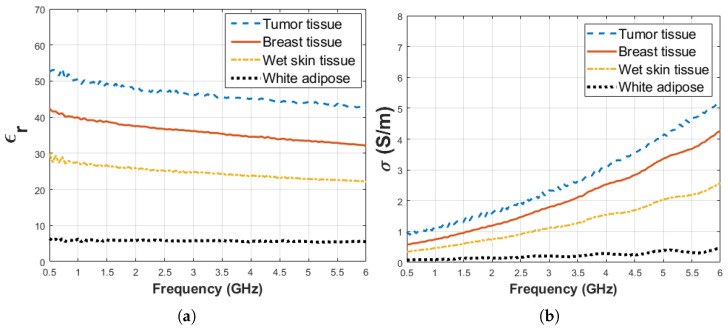
Median dielectric property measurement results between 0.5 and 6 GHz collected from various rat tissues including tumor, breast, skin, white adipose tissues: (**a**) relative permittivity and (**b**) conductivity.

The animals were given an intraperitoneal injection for anesthesia composed by mixing 80 mg kg${}^{-1}$ ketamine and 10 mg kg${}^{-1}$ xylazine. After the animal completely fell unconscious, peritoneal skin was incised, and dielectric measurement data was collected. The tumor was located between the second and fourth breast tissues as indicated with 13 in Figure 1b. The tumor had an elliptical shape and dimensions were 25.22 mm, 14.65 mm 4.4 mm for width, height and depth, respectively. Dielectric properties of thebreast tissues, wet skin from the abdomen and white adipose tissue from the nape as indicated with 14 in Figure 1c were measured on the control animal. Five dielectric property measurements were taken from each measurement point for every sample as shown in Figure 2. The median value of five measurements was selected to represent dielectric property. The average temperature of the samples was 29.5 ± 1.5 ${}^{{}^{\circ}}$C. When the measurement process was completed, the animals were immediately euthanized. The tumor was resected for pathological analysis and the breast tissues was removed as a bloc with skin attached for sensing depth analysis. The experiments were conducted in accordance with the ethical regulations approved by Bogazici University Animal Local Ethics committee. In vivo relative permittivity and conductivity measurement results of breast, wet skin, white adipose and tumor tissues are given in Figure 3a,b, respectively. The standard deviation from the mean of five measurements for each tissue sample was calculated in the frequency range of 0.5–6 GHz with 101 points. The maximum standard deviation of dielectric property was listed in Table 1 for each tissue sample along with corresponding frequency point.

## 3. Materials and Methods

In the following section, the details of experiment setup, preparation of two double-layered sample configurations and applied dielectric property measurement protocol in detail are explained.

### 3.1. Experiment Setup

**Figure 4:**
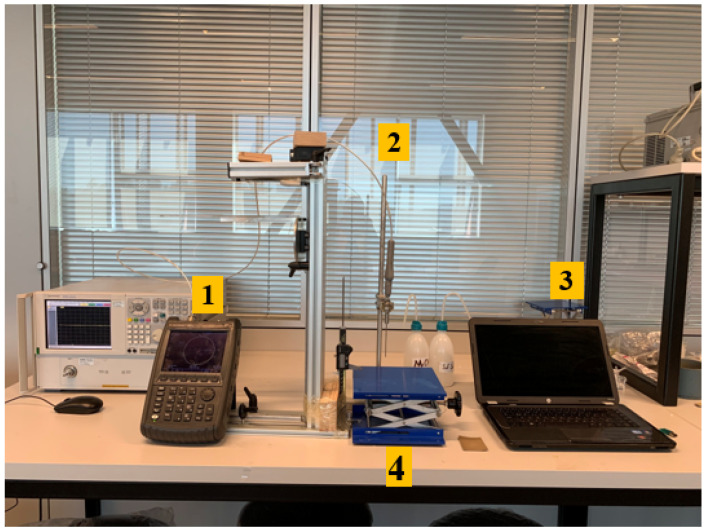
Experimental setup includes (1) Agilent FieldFox N9923A 6GHz RF Vector Network Analyzer (VNA), (2) Agilent Dielectric Slim Form open-ended coaxial probe, (3) Agilent 85070E software, external computer, (4) an adjustable stand and a digital caliper.

The experiment setup is composed of an Agilent FieldFox N9923A 6 GHz RF Vector Network Analyzer (VNA) (Santa Clara, CA, USA), Agilent Dielectric Slim Form open-ended coaxial probe (2.2 mm aperture diameter) (Santa Clara, CA, USA), Agilent 85070E dielectric property measurement software (Santa Clara, CA, USA), external computer, an adjustable stand, a digital caliper and two double-layered configuration samples. The setup is shown in Figure 4 and the function of each equipment is marked with 1 to 4 given in the figure:

1. The function of the VNA was to retrieve the S parameter responses from the probe. To eliminate temperature drift errors caused by device and cable connections, the VNA was turned on four hours prior to measurements. The frequency range was set from 0.5 to 6 GHz with 55 MHz increments which is a sufficient range for the biological tissue studies.
2. The 2.2 mm-diameter open-ended coaxial probe was used in this experiment. It is a widely preferred dielectric property measurement tool due to its wideband properties and non-destructive measurement features [9,12,17,18]. Additionally, the position of the probe was fixed and remained in the same position during the entire experiment to avoid errors caused by cable movement.
3. Agilent 85070E software was used to calculate the complex permittivity from the retrieved S parameters. The software requires guided “three standards” calibration steps in the order of open circuit, short circuit and a broadband load. In our case, the probe tip was terminated with open air, a conductive textile and distilled water with known temperature, respectively.
4. The sample location was defined via an adjustable stand and a caliper. The top plate of the adjustable stand was a stainless steel structure and the thickness of plate was 12 mm. The adjustable stand was used to move the sample up and down while the caliper measures the position of the stand during the experiment. The probe tip distance from layers was measured via Mitutoyo absolute digimatic caliper 0–150 mm with 0.01 mm digital step size.

**Figure 5:**
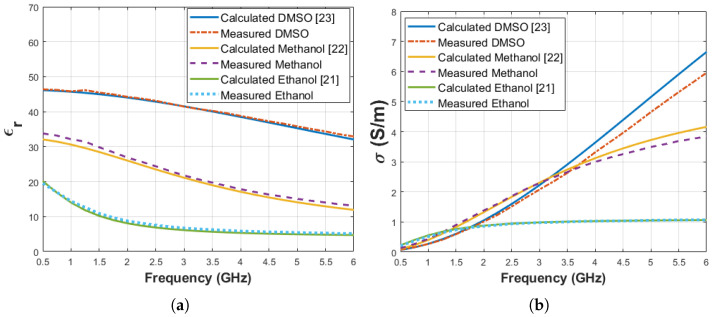
Comparison of known materials (ethanol, methanol and DMSO) dielectric properties calculated from Debye parameters obtained from the literature and measured by the experiment setup: (**a**) relative permittivity, (**b**) conductivity.

System validation was performed with three known materials: ethanol ($\epsilon_{r}$ = 8.73 and σ = 0.86 S/m at 2 GHz), methanol ($\epsilon_{r}$ = 26.86 and σ = 1.38 S/m at 2 GHz) and Dimethyl Sulfoxide (DMSO) ($\epsilon_{r}$ = 44.23 and σ = 1.02 S/m at 2 GHz). The measurements were taken when the average temperature of materials was 28.3 ± 0.2 ${}^{{}^{\circ}}$C. The measurement results were compared with calculated dielectric properties based on the Debye parameters from literature [21,22,23]. The temperatures of the materials were 29.5 ${}^{{}^{\circ}}$C in the literature. The measured and calculated dielectric properties are shown in Figure 5. Also, maximum difference between measured and calculated dielectric properties from the Debye parameters given in the literature are listed in Table 2 at the corresponding frequency points.

### 3.2. Sample Configuration

**Figure 6:**
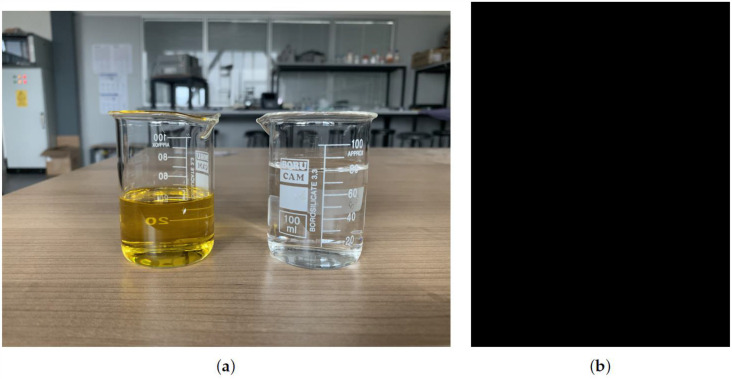
Sample configuration: (**a**) olive oil (left) and Triton X-100 (right) were used as second layer liquids and (**b**) double-layered configuration with rat breast tissue and olive oil.

The prepared two double-layered configurations consisted of rat breast and skin tissues as the first layer and triton X-100 and olive oil as the second layer. The first sample configuration included rat breast tissue and olive oil. The second sample configuration was composed of rat wet skin tissue and triton X-100. The liquids used to form the second layer are shown in Figure 6a (from left to right: olive oil and Triton X-100). The tissues were removed from 359 days old, 285 g Sprague-Dawley rat. The thickness of the rat tissue was 2.5 mm, and each sample was resected approximately with 3.5 cm-diameter. The measurements were performed within 1 h following the excision of rat tissues. A sample of the double-layered configuration composed of rat breast tissue-olive oil is given in Figure 6b.

**Figure 7:**
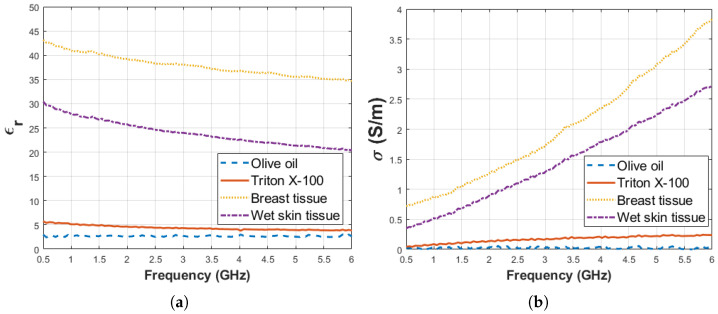
Median of dielectric properties obtained from pure materials olive oil, Triton X-100 and ex vivo rat tissues: (**a**) Relative permittivity and (**b**) conductivity.

Before starting the experiment, dielectric property measurement was performed for pure materials; that is, triton X-100 and olive oil. Also, dielectric property measurements were collected from ex vivo rat tissues. Five measurements were taken for every sample and median of the measurement results are given in Figure 7a,b for relative permittivity and conductivity, respectively. The average temperature of pure materials was 27.5 ± 0.5 ${}^{{}^{\circ}}$C. The standard deviation from the mean of five measurements for pure materials (olive oil, triton X-100, ex vivo breast and wet skin tissues) was calculated in the frequency range of 0.5–6 GHz with 101 points. The maximum standard deviations of dielectric properties are listed in Table 3 for each pure material at corresponding frequency points.

### 3.3. Measurement Protocol

**Figure 8:**
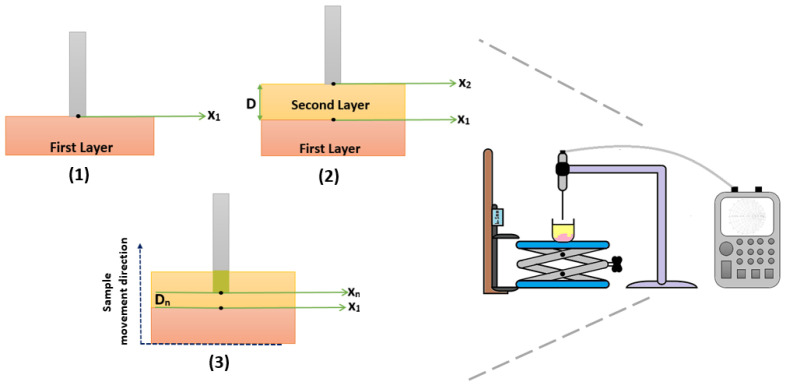
Measurement protocol for analyzing sensing depth with double-layered sample configuration. (1) X${}_{1}$ position was determined based on the thickness of the first layer. (2) X${}_{2}$ position was determined based on the probe tip distance from the first layer (D) was calculated (X${}_{2}$ − X${}_{1}$). (3) Double-layered sample configuration was gradually moved up to analyze the dielectric property change and sensing depth of the probe.

The applied measurement protocol for sensing depth characterization of the 2.2 mm open-ended coaxial probe shown in Figure 8. To eliminate potential error due to calibration degeneration, the open-ended coaxial probe, the probe holder, VNA, adjustable stand and caliper were placed in a fixed position and the same position was maintained during the measurement process. The distance between the probe tip and sample was adjusted by moving the adjustable stand; that is, the sample under test. Since a special measurement tank was not employed to simplify the measurement procedure, the sample movement was tracked by measuring the adjustable stand height. To do so, the caliper jaw was firmly attached to adjustable stand as shown in Figure 8. The protocol is described as follows:

- Steps in Figure 8(1)
  − The first layer (rat tissues) was laid flat at the bottom of a glass beaker. Please note that the tissues were fixed at the bottom of beakers by using a double-sided tape.
  − The beaker was placed on the platform of the adjustable stand and slowly moved towards the probe tip.
  − While the adjustable stand was moving towards the probe tip, the change in the S parameter response from the VNA was monitored. When a response significantly different from the open air; that is, S parameter response of the probe converging to that when the probe was terminated with the tissue, was observed, it was confirmed that the first layer was touching to the probe tip. At this point, the measured value on the caliper was recorded as X${}_{1}$ position. Please note that a dielectric property measurement was not performed at this step of the protocol to prevent any potential damage to tissue surface due to pressure.
  − The information of X${}_{1}$ position reduces misleading sensing depth measurements caused by the probe pressure to the rat tissues.
- Steps in Figure 8(2)
  − The adjustable stand was moved down to place the second layer liquid.
  − Without shifting the location of the sample, the liquids (Triton X-100 or olive oil) were gently added with a Pasteur pipette.
  − Next, the double-layered sample was moved towards the probe tip.
  − When the surface of the second layer was in full contact with the tip of the probe, the measured value on the caliper was recorded as X${}_{2}$ position.
  − The difference between X${}_{1}$ and X${}_{2}$ positions provides the distance “D” which is the distance from the probe tip to the surface of the first layer.
  − Knowledge of the distance D allows us to determine the increment size of the adjustable stand movement.
- Steps in Figure 8(3)
  − The double-layered sample was gradually moved up allowing the probe to immerse into the second layer. The dielectric properties were measured in each position (X${}_{n}$) at different depths (D${}_{n}$).
  − The measurements were concluded when the probe tip arrived at the X${}_{1}$ position, which is the surface of rat tissues.

## 4. Results

This section demonstrates the sensing depth analysis of the 2.2 mm-diameter open-ended coaxial probe based on the dielectric properties acquired from the two different double-layered configurations. The analysis was conducted by selecting five frequency points. The percent change in the measured dielectric properties of the double-layered configurations were analyzed at each frequency point with respect to the probe tip distance from the first layer (D${}_{n}$).

### 4.1. Sensing Depth Analysis: Rat Breast Tissue-Olive Oil Configuration

**Figure 9:**
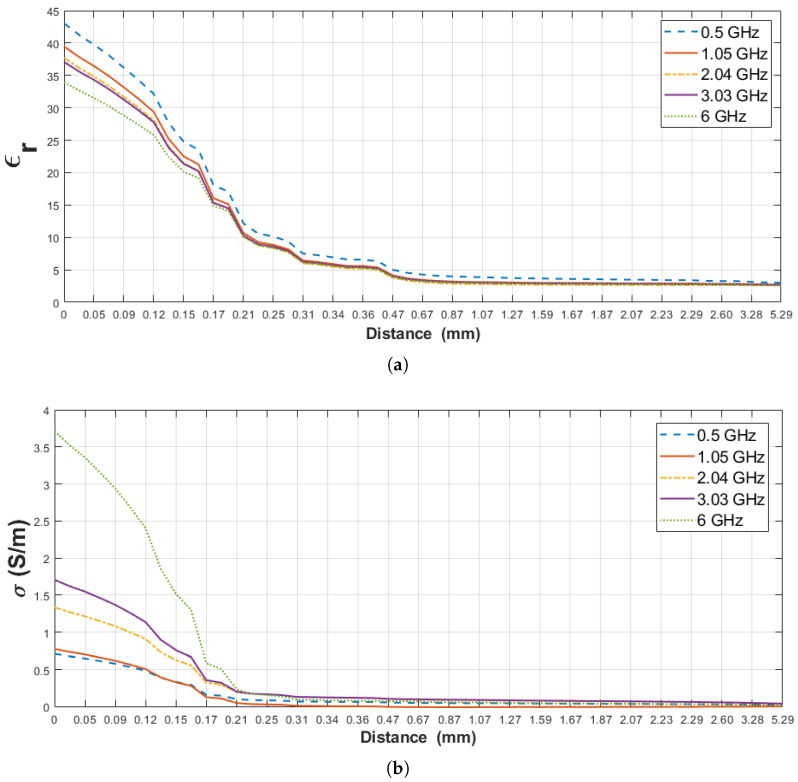
Measured dielectric properties of the double-layered rat breast tissue-olive oil configuration: (**a**) Relative permittivity, (**b**) conductivity as a function of probe tip distance from first layer (D${}_{n}$) at 0.5, 1.05, 2.04, 3.03 and 6 GHz frequencies.

The relative permittivity and conductivity of pure materials (rat breast tissue and olive oil) used for this scenario are listed in Table 4. Moreover, the change in measured dielectric properties of the rat’s breast tissue-olive oil configuration is plotted in Figure 9 for the selected five frequency points, 0.5, 1.05, 2.04, 3.03 and 6 GHz, against probe distance from the surface of the rat breast tissue. When the distance on the graphs is equal to zero, it refers to the probe tip touching on the rat breast tissue and the obtained values are compatible with the pure material dielectric properties. In Figure 9b, for distances smaller than 0.17 mm the dominant material is rat breast tissue and the largest conductivity measured for the largest frequency. Around 0.17 mm the dominant material starts to become olive oil. Since the olive oil conductivity is very small, the system uncertainties start to be evident and same frequency-conductivity trend becomes less apparent. Furthermore, the relative permittivity and conductivity of the olive oil were very low ($\epsilon_{r}$ = 2.45 and σ = 0.0162 S/m at 2.04 GHz) and the reported error of the commercial systems is 5% [24]. In Figure 9, the results were shown with polylines due to the dielectric property of selected materials, system error and small incremental steps (approximately 0.02 mm). For analysis purposes, we investigated the dielectric property alteration of the double-layered configuration with respect to five percent change points: 5%, 10%, 20%, 80% and 90%. First, the increase in the relative permittivity and conductivity of olive oil with respect to pure olive oil measurement was examined based on these percentage values. Second, the decrease in relative permittivity and conductivity of breast tissue with respect to breast tissue measurement was analyzed based on same percentage changes. These percentage values were selected according to sensing depth analysis in the following literature. The dielectric permittivity of intervening liquid was small in our experiments, therefore we selected 5% changes according to the defined histology region with system uncertainty in [18]. 10% changes in dielectric property of alcohol samples was also determined as an accepted threshold for sensing depth of 2.2 mm-diameter probe [16]. The effective penetration depth was described as a 20% difference between expected and measured dielectric property in [15]. Approximately 90% of dielectric property for the intervening liquid was pointed out for sensing depth analysis of 2.2 mm-diameter probe [17]. 80% and 90% changes were also selected as complementary to 20% and 10% changes, respectively.

**Figure 10:**
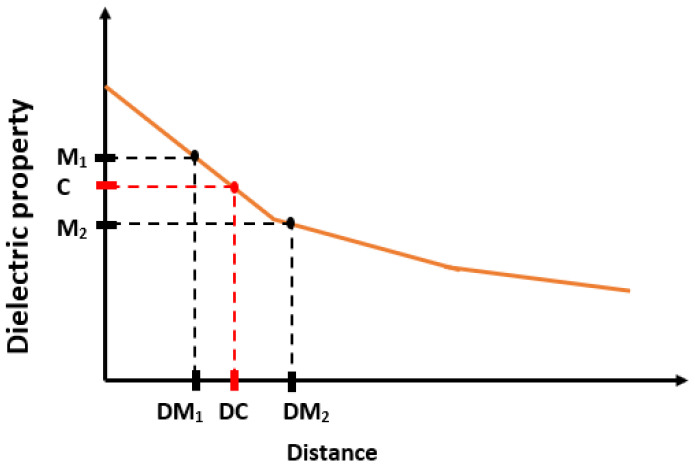
The distance (DC) was estimated for calculated dielectric property (C) based on percentage change using measured consecutive dielectric properties (M${}_{1}$ and M${}_{2}$) and their distances (DM${}_{1}$ and DM${}_{2}$).

The aim here is to find the distances where 5%, 10%, 20%, 80% and 90% change in the dielectric property of pure materials (olive oil and rat breast tissue) are observed. Since the dielectric properties of the pure materials are known, as shown in Table 4, the percentage changes mentioned above can be mathematically computed. On the other hand, the measured dielectric properties obtained from the distances (D${}_{n}$) may not correspond to the expected percentage change. Therefore, an error margin is defined such that if the measured dielectric property from a certain distance (D${}_{n}$) varies within 1% of the expected percentage change, then that distance is accepted as the distance where the expected percentage change occurs. Otherwise, if the measured dielectric property is larger or smaller than 1% of the expected percentage change, the distance corresponding to the expected percentage change is determined by using the estimation procedure given in [15]. Suppose that M${}_{1}$ and M${}_{2}$ are the measured dielectric properties from the distances DM${}_{1}$ and DM${}_{2}$, respectively and the expected percentage change in dielectric properties (C) falls between M${}_{1}$ and M${}_{2}$ (see Figure 10). To obtain the distance (DC) corresponding to the expected percentage change, a linear line is defined using the points (M${}_{1}$, M${}_{2}$, DM${}_{1}$ and DM${}_{2}$). The distance (DC) corresponding to the expected change in dielectric property is calculated from the linear line equation.

First, the percent change in the measured dielectric property of the double-layered configuration was analyzed regarding the increase in the dielectric property of the second layer (olive oil). The percent change in the measured dielectric properties of the configuration and the corresponding distances (D${}_{n}$) of the probe tip at five frequency points are listed in Table 5. Table 5 indicates 5% increment in relative permittivity and conductivity of the configuration from 1.36 to 3.28 mm and from 4.8 to 5.1 mm probe tip distances from the first layer (D${}_{n}$), respectively, at the five frequency points. 10% increase in the measured relative permittivity and conductivity was observed from 0.87 to 2.31 mm and from 4.4 to 5.2 mm and 20% increase from 0.67 to 1.67 mm and from 0.37 to 4.6 mm, respectively. As the probe tip approached to the first layer, the effect of this layer became dominant resulting in an 80% increase in the measured relative permittivity between 0.40 and 0.41 mm and measured conductivity between 0.36 and 2.6 mm distances. Furthermore, 90% increase in relative permittivity is seen between 0.38 and 0.42 mm and similar increase in conductivity between 0.35 and 2.29 mm is observed indicating that the probe tip was located at the region where the measurements reflect the dominance of the first layer.

Secondly, the percent change in the measured dielectric property of the double-layered configuration was evaluated with respect to the decrease in the dielectric property of the first layer (rat breast tissue). In Table 6, the measured relative permittivity and conductivity of the double-layered configuration decreased by 5% at 0.02 mm and at 0.03 mm probe tip distances respectively, for the given five frequency points. 10% decrease in permittivity from 0.06 to 0.07 mm and same percent decrease in conductivity is observed at 0.05 mm. Likewise, 20% decrease in the measured relative permittivity and conductivity is seen at 0.1 mm and at 0.09 mm probe tip distances, respectively. As the probe tip moved away from the first layer, the effect of the second layer happened to be more dominant, leading to 80% decrease in measured relative permittivity between 0.27 and 0.29 mm probe tip distance and 80% decrease in measured conductivity between 0.17 and 0.18 mm. In addition, the measured relative permittivity and conductivity decreased by 90% between 0.47 and 0.67 mm probe distance and between 0.18 and 0.31 mm respectively, indicating that the second layer has more effect in this region.

### 4.2. Sensing Depth Analysis: Rat Wet Skin Tissue—Triton X-100

**Figure 11:**
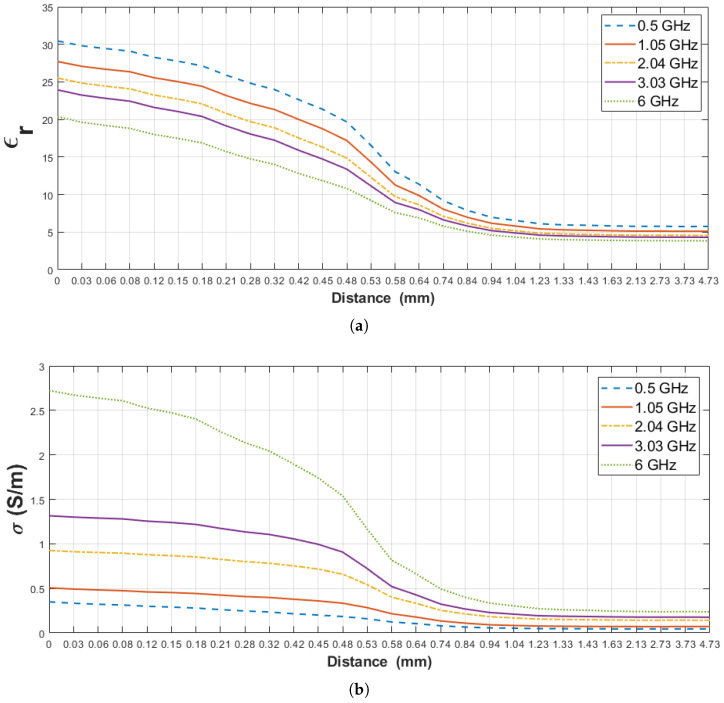
Retrieved dielectric properties of the double-layered rat wet skin tissue and triton X-100 configuration: (**a**) Relative permittivity, (**b**) conductivity as a function of probe’s distance from first layer (D${}_{n}$) at 0.5, 1.05, 2.04, 3.03 and 6 GHz.

The relative permittivity and conductivity of pure materials (rat wet skin tissue and triton X-100) used for this scenario are listed in Table 7. Furthermore, similar to the previous section, the sensing depth of the probe was analyzed based on measured dielectric property change of both layers.Triton X-100 is not expected to interact with the tissue in a short period of time at room temperature [25]. In Figure 11, the change in dielectric property of rat wet skin tissue–triton X-100 double-layered configuration is given for five frequency points against the probe distance from the surface of the first layer (D${}_{n}$). The percent change in the measured dielectric property of the rat wet skin tissue-triton X-100 configuration was first analyzed based on the increase in dielectric property of the second layer (triton X-100). Table 8, denotes the 5%, 10%, 20%, 80% and 90% increment points in the measured dielectric properties and their corresponding distances (D${}_{n}$) at five frequency points. From the table, 5% increase in the measured relative permittivity at 1.23 mm and same percent increase in conductivity is observed between 1.33 and 1.43 mm probe tip distances. 10% and 20% increase in the measured relative permittivity are occurred at 1.11 mm and at 0.94 mm, respectively. Also, 10% and 20% increase in conductivity are observed from 1.23 to 1.33 mm and from 1.04 to 1.23 mm, respectively. Due to the close proximity of the probe to the first layer, 80% and 90% increase in the relative permittivity was observed between 0.64 and 0.69 mm probe tip distance and between 0.61 and 0.66 mm, respectively. Similarly, 80% and 90% increase conductivity was seen between 0.81 and 0.74 mm distances and between 0.72 and 0.78 mm, respectively. These values clearly show that the first layer was dominant with these distances (D${}_{n}$).

The second analysis was based on the percent change in the measured dielectric property of double-layered configuration with respect to the decrease in dielectric property the first layer (rat wet skin tissue). Table 9, show 5% decrease in measured relative permittivity and conductivity of the double-layered configuration from 0.06 to 0.08 mm and from 0.03 to 0.12 mm, respectively. Similarly, 10% decrease in relative permittivity and conductivity from 0.12 to 0.18 mm and from 0.08 to 0.21 mm probe distances, respectively, and 20% decrease from 0.18 to 0.32 mm and from 0.18 to 0.42 mm probe distances, respectively. Finally, 80% decrease the relative permittivity was observed between 1.04 and 1.33 mm and 80% decrease in conductivity between 0.74 and 0.94 mm. The high percentage decrease is due to the dominant nature of the second layer since the probe tip moved further away from the first layer. It should be noted the 90% decrease in the dielectric property was not calculated since the dielectric property of triton X-100 is higher than the calculated values.

## 5. Discussion

**Figure 12:**
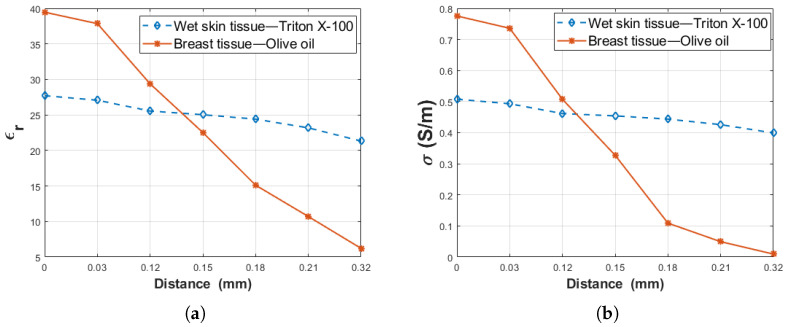
The comparison dielectric property alteration of rat wet skin tissue-triton X-100 and rat breast tissue-olive oil as a function of probe tip distance (D${}_{n}$): (**a**) relative permittivity and (**b**) conductivity.

This study was performed to examine how various adnexal structures that can be formed on wet skin and around breast tissues can affect measurements. Moreover, two double-layered configurations were prepared to determine the effect of the dielectric property contrast between layers on the probe’s sensing depth. The first configuration was composed of rat breast tissue ($\epsilon_{r}$ = 39.35 and σ = 0.8607 S/m at 1.05 GHz) and olive oil ($\epsilon_{r}$ = 2.73 and σ = 0.0034 S/m at 1.05 GHz). In the second configuration, rat wet skin tissue ($\epsilon_{r}$ = 26.86 and σ = 0.4812 S/m at 1.05 GHz) and triton X-100 ($\epsilon_{r}$ = 5.11 and σ = 0.0722 S/m at 1.05 GHz) were used. For the first configuration, the difference between the layers are 36.62 units for relative permittivity and 0.8573 S/m for conductivity. The difference between the layers for the second configuration are 21.75 units for the relative permittivity and 0.409 S/m for conductivity. From these results, the difference of relative permittivity between the layers decreased 40% in the second configuration. Similarly, the difference of conductivity was declined 52% in the second configuration. To analyze the impact of contrast between the layers, the dielectric property change of the two configurations was compared at the frequency point of 1.05 GHZ as given at Figure 12. The dielectric properties of both configurations were plotted in the distance range of 0–0.32 mm (D${}_{n}$). Since the most important change was observed at the close proximity of the probe tip, the most precise increment was implemented at this interval even though manual measurements were performed. Therefore, the distances are only the same at this interval for both configurations. As seen in Figure 12, since rat breast tissue-olive oil has a larger dielectric property difference a significant change was observed between 0–0.32 mm, the rat wet skin tissue-triton X-100 shows a more gradual change due to the relatively smaller dielectric property difference between the layers. In [18], the dielectric property contrast between first and second layers of the double-layered configuration were examined independent of frequency, and similar results were also observed.

**Figure 13:**
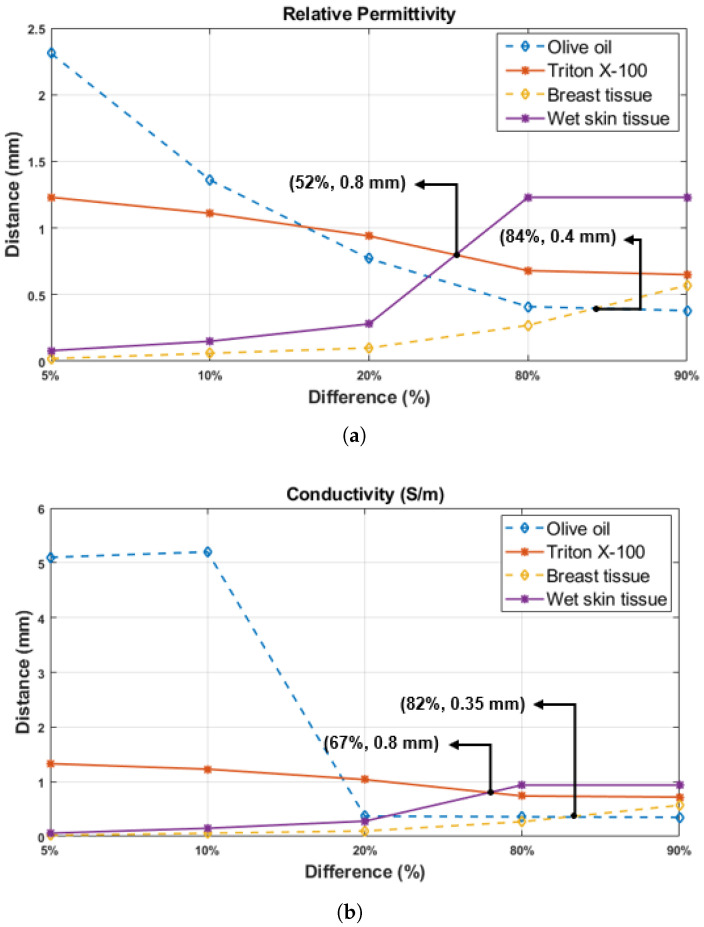
The probe tip distances from rat tissue layers (D${}_{n}$) are shown as a function of five percentage change points in dielectric property of the materials used for double-layered configurations (olive oil, triton X-100, rat breast and wet skin tissue): (**a**) relative permittivity and (**b**) conductivity.

In addition, the dielectric property based on the percentage change was plotted using the data from Table 5, Table 6, Table 7, Table 8 and Table 9 at the result Section 4.1 and Section 4.2. The Figure 13 demonstrates the probe tip distances from the first layer (D${}_{n}$) as a function of dielectric property percentage change in double-layered configuration at the 1.05 GHz frequency point. As seen in Figure 13, 20%, 80%, and 90% changes occur at smaller distances for rat breast tissue and olive oil, while the same percentage changes are observed at larger distances for the rat wet skin tissue and triton X-100. Furthermore, the intersection between wet skin tissue and triton X-100 alterations is observed at the 52% relativity permittivity change point and 0.8 mm probe tip distance from the first layer (D${}_{n}$) as shown in Figure 13a. Similarly, intersection for the rat breast tissue and olive oil alteration is noticed at the 84% change point and 0.4 mm distance. For the conductivity, as given in Figure 13b, while the intersection of rat breast tissue and olive oil happens at 82% change and 0.35 mm distance point, the intersection of rat wet skin tissue and triton X-100 alterations happens at 67% change and 0.8 mm distance. The percentage change of relative permittivity for both configurations were examined in the frequency range of 0.5–6 GHz and it was concluded that the results are similar to Figure 13a. However, the change of the percentage based on conductivity of olive oil in the same frequency range is different from each other, contrary to the triton X-100 alteration. This difference between conductivity changes can be caused by the very low conductivity of olive oil. Although the small conductivity of olive oil caused difficulty in percentage analysis, the conductivity at 1.05 GHz showed percentage change similar to the relative permittivity variations. As also explained in the study [18], conductivity shows ambiguity at sensing depth studies. Therefore, relative permittivity changes should be prioritized since they give more precise results. When measuring the relative permittivity of the skin or breast tissues, it was examined that a thin layer (such as collagen, white adipose or subcutaneous tissue) formed on targeted tissue, even if it has 0.4–0.8 mm thickness, can confound the results between 52% and 84%. To avoid these errors, it is important to establish a-priori information regarding the tissue or sample of interest. For rat tissues, even though the animal’s anatomy is known well, small layers that cannot be recognized by the naked eye can have a significant impact on the results. For more accurate measurement results, the location of tissue to be measured must be precisely determined and measurements must be taken from areas surrounding the target point as well for cross-validation purposes. Moreover, the tissue can be scratched lightly before taking the measurement to remove the layer that may be formed on the target tissue. Therefore, there is a need to formulate a protocol for experiments to reduce errors caused by tissue heterogeneity. Another option is to catalogue sample specific sensing depth information and use the appropriate information in machine learning algorithms or choose the probe with the appropriate aperture size. Alternatively, the measured dielectric properties with known probe sensing depth can be used for heterogeneity grading or tumor grading.

## 6. Conclusions

Acquiring the dielectric property of biological tissue accurately is a prerequisite for microwave detection, diagnosis and treatment methods in the medical field. Even though the open-ended coaxial probe method provides essential advantages, the technique still suffers from having high measurement error and low repeatability. Rats are commonly preferred experimental animals since they have numerous advantages. The studies for breast cancer with rat models are significant due to the characteristic of rats, which is the extent of having more hormone responsiveness with histopathology and premalignant stages similar to the human breast cancer model [26]. To avoid acquiring incorrect data caused by a heterogeneous formation beneath the rat’s pelt, dielectric measurement must be implemented rigorously. In addition, leaking of milk from the gland and body fluids or bleeding during the experiment can confound the measurement. To analyze the impact of any layer formed over the rat tissues (breast and wet skin) during the measurements, we performed sensing depth analysis of the 2.2 mm-diameter open-ended coaxial probe.

To this end, this work investigates the sensing depth of 2.2 mm-diameter open-ended coaxial probe for animal experiment. Unlike previously reported studies, the experiments consisted of a simple measurement setup without forming a special experiment setup tank. The double-layered samples included rat breast and wet skin tissue as a first layer and liquids (olive oil and triton X-100) with relatively low dielectric properties added as second layers. The difference of relative permittivity and conductivity between the layers decreased 40% and 52%, respectively, in the second configuration. Liquids with low dielectric properties provides substantial insight for potential change in measured dielectric properties since the liquids mimic the effect of adipose tissue which can cover the top of the healthy tissues and tumors. Five percent thresholds in dielectric property changes; 5%, 10%, 20%, 80% and 90% were tracked to define the sensing depth at five frequency points. The wide change from 5% to 90% increase in relative permittivity was taken place for the olive oil between 3.28 to 0.42 mm probe tip distance from the breast tissue at 0.5 GHz. Similarly, from 5% to 90% increase in conductivity for the olive oil was calculated in the distance range of 5.2–0.38 mm at 1.05 GHz. The dielectric property alteration of triton X-100 was in the range of 1.43–0.61 mm probe tip distance. Furthermore, the percent decrease in dielectric properties for rat breast and skin tissues was in the distance range of 0.02–1.33 mm. We can also state that a membrane layer (between 0.4–0.8 mm thickness) on the wet skin tissue and breast tissue will potentially affect the measurement results of dielectric property by 52–84%. These findings also suggests the layer that is in immediate contact with the probe tip will have the most significant effect on dielectric property measurements.

## Figures and Tables

**Table 1:** Maximum standard deviation from the mean of five measurements for rat tissues (tumor, breast, wet skin and white adipose) over the frequency range of 0.5–6 GHz.

Tissue Types	Relative Permittivity		Conductivity (S/m)	
	Standard Deviation	Frequency (GHz)	Standard Deviation	Frequency (GHz)
Tumor Tissue	0.68	0.72	0.04	5.73
Breast Tissue	3.23	0.5	0.34	6
Wet Skin Tissue	3.16	0.5	0.40	6
White Adipose	0.64	4.46	0.02	5.95

**Table 2:** Maximum dielectric property difference between measurements and Debye model calculation for known materials (ethanol [21], methanol [22] and DMSO [23]) used for system validation.

Materials	Difference for $\epsilon_{r}$	Frequency (GHz)	Discrepancy for σ (S/m)	Frequency (GHz)
Ethanol	0.8	1	0.04	1.25
Methanol	1.8	1.25	0.31	6
DMSO	0.9	6	0.69	6

**Table 3:** Maximum standard deviation of five measurements over the frequency range of 0.5–6 GHz for pure materials (olive oil, triton X-100, breast and wet skin).

Tissue Types	Relative Permittivity		Conductivity (S/m)	
	Standard Deviation	Frequency (GHz)	Standard Deviation	Frequency (GHz)
Olive oil	0.13	4.02	0.02	3.59
Triton X-100	0.07	0.88	0.01	3.59
Breast tissue	1.13	5.84	0.2	5.73
Wet skin tissue	1.97	6	0.3	6

**Table 4:** The relative permittivity and conductivity of pure materials (rat breast tissue and olive oil) at five frequency points.

Frequency (GHz)	Relative Permittivity		Conductivity (S/m)	
	Rat Breast Tissue	Olive Oil	Rat Breast Tissue	Olive Oil
0.5	43.2	2.59	0.72	0.0103
1.05	40.8	2.53	0.86	0.0055
2.04	39.1	2.45	1.30	0.0162
3.03	38.0	2.50	1.76	0.0170
6	34.7	2.45	3.83	0.0094

**Table 5:** Specified increase levels for retrieved relative permittivity and conductivity of double-layered rat breast tissue and olive oil configuration and the corresponding distances (D${}_{n}$) at 0.5, 1.05, 2.04, 3.03 and 6 GHz frequency points.

Freq (GHz)	Distance (mm) for Rat Breast Tissue-Olive Oil									
	Relative Permittivity					Conductivity (S/m)				
	5%	10%	20%	80%	90%	5%	10%	20%	80%	90%
0.5	3.28	2.31	1.67	0.44	0.42	4.8	4.4	3.6	1.77	1.67
1.05	2.31	1.36	0.77	0.41	0.38	5.2	5.1	0.37	0.36	0.35
2.04	1.36	0.87	0.67	0.4	0.38	4.9	4.5	3.5	1.77	1.62
3.03	2.29	1.36	0.77	0.4	0.38	5.0	4.6	3.9	2.07	1.87
6	2.31	1.36	0.77	0.41	0.38	5.1	4.9	4.6	2.6	2.29

**Table 6:** Specified decrease levels for retrieved relative permittivity and conductivity of double-layered rat breast tissue and olive oil configuration and the corresponding distances (D${}_{n}$) at 0.5, 1.05, 2.04, 3.03 and 6 GHz frequency points.

Freq (GHz)	Distance (mm) for Rat Breast Tissue-Olive Oil									
	Relative Permittivity					Conductivity (S/m)				
	5%	10%	20%	80%	90%	5%	10%	20%	80%	90%
0.5	0.02	0.06	0.1	0.29	0.67	0.03	0.05	0.09	0.18	0.31
1.05	0.02	0.06	0.1	0.27	0.57	0.03	0.05	0.09	0.17	0.18
2.04	0.02	0.06	0.1	0.27	0.47	0.03	0.05	0.09	0.18	0.31
3.03	0.02	0.06	0.1	0.28	0.57	0.03	0.05	0.09	0.17	0.23
6	0.02	0.07	0.1	0.29	0.57	0.03	0.05	0.09	0.17	0.21

**Table 7:** The relative permittivity and conductivity of pure materials (rat wet skin tissue and triton X-100) at five frequency points.

Frequency (GHz)	Relative Permittivity		Conductivity (S/m)	
	Rat Wet Skin Tissue	Triton X-100	Rat Wet Skin Tissue	Triton X-100
0.5	30.5	5.8	0.33	0.05
1.05	27.7	5.1	0.49	0.07
2.04	25.5	4.6	0.91	0.14
3.03	23.9	4.3	1.30	0.18
6	20.4	3.8	2.67	2.41

**Table 8:** Specified increase levels for retrieved relative permittivity and conductivity of double-layered rat wet skin tissue and triton X-100 configuration and the corresponding distances (D${}_{n}$) at 0.5, 1.05, 2.04, 3.03 and 6 GHz frequency points.

Freq (GHz)	Distance (mm) for Rat Wet Skin Tissue-Triton X-100									
	Relative Permittivity					Conductivity (S/m)				
	5%	10%	20%	80%	90%	5%	10%	20%	80%	90%
0.5	1.23	1.11	0.94	0.69	0.66	1.33	1.23	1.04	0.74	0.72
1.05	1.23	1.11	0.94	0.68	0.65	1.33	1.23	1.04	0.76	0.74
2.04	1.23	1.11	0.94	0.67	0.64	1.43	1.23	1.04	0.74	0.72
3.03	1.23	1.11	0.94	0.66	0.63	1.43	1.23	1.04	0.75	0.73
6	1.23	1.11	0.94	0.64	0.61	1.43	1.33	1.23	0.81	0.78

**Table 9:** Specified decrease levels for retrieved relative permittivity and conductivity of double-layered rat wet skin tissue and triton X-100 configuration and the corresponding distances (D${}_{n}$) at 0.5, 1.05, 2.04, 3.03 and 6 GHz frequency points.

Freq (GHz)	Distance (mm) for Rat Wet Skin Tissue-Triton X-100							
	Relative Permittivity				Conductivity (S/m)			
	5%	10%	20%	80%	5%	10%	20%	80%
0.5	0.08	0.18	0.32	1.33	0.03	0.08	0.18	0.84
1.05	0.08	0.15	0.28	1.23	0.06	0.15	0.28	0.94
2.04	0.08	0.15	0.21	1.04	0.12	0.21	0.42	0.94
3.03	0.08	0.12	0.21	1.04	0.12	0.21	0.42	0.84
6	0.06	0.12	0.18	1.23	0.08	0.15	0.28	0.74

## Data Availability

Not applicable.
